# What Interventions Focused on Physical Activity Could Improve Postpartum Depression Symptoms? An Overview of Systematic Reviews with Meta-Analysis

**DOI:** 10.3390/healthcare13121419

**Published:** 2025-06-13

**Authors:** Álvaro Campos-Marin, Cristina García-Muñoz, Javier Matias-Soto, Javier Martinez-Calderon

**Affiliations:** 1 Department of Physiotherapy, Faculty of Nursing, Physiotherapy and Podiatry, University of Seville, 41009 Seville, Spain; alvcmarin@gmail.com; 2 Departamento de Ciencias de la Salud y Biomédicas, Universidad Loyola Andalucía, 41704 Sevilla, Spain; jmatias@uloyola.es; 3 CTS 1110: Understanding Movement and Self in Health from Science (UMSS) Research Group, 41009 Andalusia, Spain; jmcalderon@us.es; 4 Cochrane Rehabilitation, Functioning and Disability, London W1G 0AN, UK; 5 Departamento de Fisioterapia, Universidad de Sevilla, 41009 Sevilla, Spain; 6 Instituto de Biomedicina de Sevilla-IBiS (Hospitales Universitarios Virgen del Rocío y Macarena, CSIC, Universidad de Sevilla), 41013 Sevilla, Spain

**Keywords:** depression, exercise, physical activity, postpartum, psychiatry, yoga

## Abstract

Objectives: The objective of this overview of systematic reviews with meta-analysis was to summarize the evidence on the possible effectiveness of interventions focused on physical activity in improving and preventing postpartum depression symptoms. Methods: CINAHL (via EBSCOhost), Embase, Epistemonikos, PsycINFO, PubMed, Scopus, SPORTDiscus (via EBSCOhost), and the Cochrane library were searched from inception to 19 February 2025. The methodological quality of the included reviews was assessed with AMSTAR 2. The degree of overlap between meta-analyses was calculated. Results: A total of eight systematic reviews were included. Overall, the included meta-analyses showed favorable results regarding the effects of physical activity on postpartum depression symptoms. Considering specific physical activity modalities, the largest number of meta-analyses focused on aerobic exercise, yoga, or multimodal exercise. In all three cases, most meta-analyses found that aerobic exercise, yoga, and multimodal exercise could be beneficial in reducing postpartum depression symptoms. Furthermore, several meta-analyses explored the effectiveness of walking, finding positive results in favor of this intervention in reducing postpartum depression symptoms. Finally, movement in water was only explored in one meta-analysis, and no differences were found between these interventions and control groups. Conclusions: Aerobic exercise, walking, yoga, and multimodal exercise programs may improve postpartum depression symptoms. Movement in water was not more effective than control groups for reducing this outcome. However, the results of our overview should be considered with caution, since important methodological and clinical implications have been discussed (e.g., lack of subgroups by prevention and treatment) and should guide the development of future systematic reviews on this topic.

## 1. Introduction

Women’s mental health is often affected during postpartum. Psychiatric symptoms and disorders such as post-traumatic stress disorder, anxiety, and depression may be common during this period. A systematic review with meta-analysis has recently found that the prevalence of post-traumatic stress disorder may reach 11% in women in Mainland China mainly during the first month postpartum [1]. Another systematic review with meta-analysis has shown that women may experience both anxiety and depression during postpartum [2], and the prevalence of these two factors may even increase over the years [3].

Depression is probably the psychological factor that has generated the greatest scientific interest in recent years in the field of obstetrics. During the postpartum period, depression causes mothers to experience strong feelings of loneliness, sadness, and grief, as well as multiple intrusive thoughts (e.g., “I’m not a good mother”) [4]. Postpartum depression could also be an important risk factor for suicide and even mortality in different countries. For example, a recent study conducted with Japanese women in the postpartum period found that the prevalence of suicidal ideation was 51.8% in women with postpartum depression compared with 3.3% in women who reported no depression during that period [5]. In addition, the risk of mortality may be higher considering postpartum depression in comparison with antepartum depression, after examining 86,551 Swedish women with a first diagnosis of perinatal depression [6].

In this context, the number of clinical trials exploring non-pharmacological strategies to reduce postpartum depression symptoms is growing. This has led to the development of numerous systematic reviews with meta-analysis aiming to synthetize and critically appraise this evidence, and some of them have focused on exploring the effects of physical activity modalities [7,8,9]. The benefits of interventions focused on physical activity for improving women’s mental health have been shown in different studies, including benefits in factors such as anxiety, depression, and sleep outcomes (e.g., insomnia) [10,11]. Specifically in the postpartum period, systematic reviews with meta-analysis have observed that some interventions focused on physical activity may reduce depression symptoms (e.g., aerobic exercise), although these positive effects have not been detected in all physical activity modalities that have been meta-analyzed (e.g., multimodal exercise programs) [7,8].

Therefore, the development of an overview of systematic reviews may shed light on those interventions focused on physical activities that might be most appropriate for improving and preventing postpartum depression symptoms based on the results provided by published meta-analyses on this topic, which could enormously help clinicians who directly work with this population. The objective of this overview of systematic reviews with meta-analysis is to summarize the evidence on the possible effectiveness of interventions focused on physical activity in improving and preventing postpartum depression symptoms.

## 2. Materials and Methods

This overview of systematic reviews with meta-analysis followed the Preferred Reporting Items for Overviews of Systematic Reviews (PRIOR) [12] statement (Supplementary File S1) and the Preferred Reporting Items for Systematic Reviews and Meta-Analyses (PRISMA) statement for abstracts [13] (Supplementary File S2). The review protocol was prospectively registered at the Open Science Framework: https://doi.org/10.17605/OSF.IO/ZYV9B.

### 2.1. Protocol Deviation

Supplementary File S3 shows the deviations from the review protocol.

### 2.2. Data Sources and Search Strategy

One co-author (JMC) searched the following e-databases from inception to 19 February 2025: CINAHL (via EBSCOhost), Embase, Epistemonikos, PsycINFO, PubMed, Scopus, SPORTDiscus (via EBSCOhost), and the Cochrane library. When possible, search filters were used to refine some searches. For example, theses and dissertations were not considered in PsycINFO. No search filters were imposed considering publication language, although only studies published in peer-reviewed journals and written in English or Spanish were considered for inclusion. The full search strategy for all e-databases is reported in Supplementary File S4. Manual searches were performed to complement the full search strategies. For this, review articles related to the topic of this overview were screened if they were retrieved by search strategies.

### 2.3. Eligibility Criteria

The PICOS (Population, Intervention, Comparison, Outcome, Study design) framework was used to build eligibility criteria [14].

Inclusion criteria:

P: Women during postpartum period. Women with or without postpartum depression at baseline were considered.

I: Interventions focused on physical activity were included. Therefore, we considered meta-analyses including studies evaluating physical exercise (e.g., aerobic exercise), mind–Departamento de Fisioterapia, Universidad de Sevilla, 41009 Sevilla, Spainbody exercise (e.g., yoga), and/or regular physical activities (e.g., walking). Meta-analyses focused on multimodal (e.g., aerobic plus resistance exercise programs) physical activity modalities were also included. Physical exercise includes planned, structured, and repetitive exercises [15]. Mind–body exercises are exercises that combine breathing exercises, meditation, and physical postures [16]. Regular physical activities are occupational, recreational, or sports activities involving the musculoskeletal system, which provoke energy expenditure, and are unplanned, unstructured, and are not repetitive [17]. We are aware that physical activity may not be the first line of treatment for women with postpartum depression and that other treatments (e.g., antidepressants) may be active during clinical trials. In these cases, these meta-analyses were also included if the review’s objective was to specifically explore interventions focused on physical activity.

C: There were no restrictions.

O: Depression symptoms evaluated with different scales (e.g., Edinburgh Postnatal Depression Scale).

S: Systematic reviews with meta-analysis of randomized clinical trials.

Exclusion criteria:

I. Meta-analysis where less than two studies were examined.
II. Abbreviated reports of Cochrane reviews and previous versions of Cochrane reviews that were included in our overview.
III. Network meta-analysis where direct estimates of comparisons of interest were not reported.
IV. Systematic reviews where meta-analyses were not performed by exercise modalities, mind–body exercises, or regular physical activities.
V. Meta-analysis where studies evaluating exercise, mind–body exercises, or regular physical activities were jointly analyzed with studies examining other types of interventions (e.g., mindfulness).
VI. Systematic reviews where we have not access to full text even after requesting them from corresponding authors via email.
VII. Protocols, theses, dissertations, and conference abstracts/proceedings.

### 2.4. Study Selection

Two co-authors (ACM and JMC) performed study selection. One co-author (ACM) used Zotero 7.0.13 Citation Management Software to include all references retrieved by e-databases and remove duplicates. ACM screened these references by title and abstract and included in a table those studies that may satisfy our inclusion criteria and were subsequently analyzed at full text. Afterward, two co-authors (ACM and JMC) independently assessed studies at full text. The percentage of agreement between ACM and JMC was calculated considering the number of items rated with the same score before pooling the results of their independent assessments. The percentage of agreement between ACM and JMC was calculated (86.15%).

### 2.5. Methodological Quality Asssessment

Two co-authors (ACM and JMS) independently used AMSTAR 2 to assess the methodological quality of systematic reviews [18]. AMSTAR 2 is composed of sixteen items that can be rated as ‘Yes’, ‘Partially Yes’, or ‘No’. Seven of these items are recommended as critical domains (items: 2, 4, 7, 9, 11, 13, 15) and an overall score is not recommended [18]. The percentage of agreement between ACM and JMS was calculated considering the number of items rated with the same score before pooling the results of their independent assessments.

### 2.6. Data Extraction

Two co-authors (ACM and JMC) independently extracted from each systematic review the following information:

I. First author and year of publication.
II. Objective original review; N total of participants; population details; number of randomized clinical trials; outcome analyzed in this overview; target intervention: (prevention or treatment).
III. Intervention characteristics: period of physical exercise (prenatal or postnatal) and physical activity modalities analyzed in this overview.
IV. Type of control group.
V. Meta-analyses of interest and the certainty of evidence evaluated with the Grading of Recommendations Assessment, Development and Evaluation (GRADE) system.

The percentage of agreement between ACM and JMC was calculated considering the number of items rated with the same score before pooling the results of their independent assessments. The factor “target intervention” was not considered when the percentage of agreement was calculated since this factor was included a posteriori. The percentage of agreement between ACM and JMC was calculated (79%).

### 2.7. Data Analysis and Synthesis

Table 1 shows all meta-analyses of interest and the characteristics of the included studies. The results of meta-analyses were also reported in the main text divided into physical activity modalities (e.g., yoga). When systematic reviews reported different meta-analyses by time-point (e.g., post-intervention and follow-up), we reported all time-points in Table 1 and the main text for transparency.

### 2.8. Overlap Between Meta-Analyses

The degree of overlap between meta-analyses was calculated. One co-author (JMC) built matrices of evidence by physical activity modality (e.g., aerobic exercise). These matrices of evidence were only developed when at least two meta-analyses examined the same physical activity modality (e.g., yoga). These matrices of evidence were needed to calculate the corrected covered area (CCA), which is essential to calculate the degree of overlap between meta-analyses [19]. The CCA refers to the area that is covered after removing the original studies the first time they are counted. The calculation of the CCA is based on three aspects: N is the total number of original studies included in the meta-analyses of interest (including original studies duplicates). Furthermore, r is the number of original studies without accounting for duplicates. Finally, c is the number of systematic reviews included in the matrix of evidence [19]. The degree of overlap can be classified as slight (CCA 0–5%), moderate (CCA 6–10%), high (CCA 11–15%), or very high (CCA > 15%) [19]. The degree of overlap was also visually represented. One co-author (CGM) developed a bar plot to depict the degree of overlap between meta-analyses for each matrix of evidence.

## 3. Results

**Figure 1:**
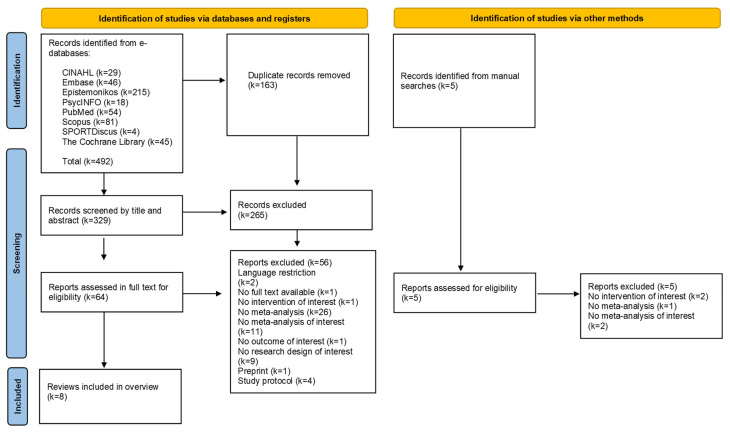
PRISMA flow diagram of studies selection.

Figure 1 shows the PRISMA flow chart with the study selection process. The list of studies analyzed at full text and the excluded studies with the reasons for exclusion is reported in Supplementary File S5. A total of eight e-databases were searched and 492 references were retrieved. After removing duplicates, 329 references were analyzed by title and abstract. Of them, 64 references were analyzed at full text, and seven systematic reviews with meta-analysis were eventually included [7,8,20,21,22,23,24]. Table 1 shows all meta-analyses of interest and the characteristics of the included studies.

**Table 1:** Characteristics of the included systematic reviews.

Study and Year of Publication	Main Characteristics of Systematic Review	Intervention Characteristics	Type of Control Groups	Meta-Analyses and Certainty of Evidence (GRADE)
Davenport et al., 2018 [7]	Objective original review: to summarize the evidence on effects of prenatal physical exercise on depression and anxiety during pregnancy and the postpartum period. N total of participants: 131.406. Population: women without contraindications to physical exercise during pregnancy. Number of randomized clinical trials: 26. Outcome analyzed in this overview: depression symptoms (not specified in inclusion criteria how they were assessed). Target intervention: treatment of depression symptoms.	Period of physical exercise: prenatal physical exercise. Physical exercise modalities analyzed in this overview: yoga.	Multiple controls were analyzed in the meta-analysis: no physical exercise or different frequency, intensity, duration, volume, and type of physical exercise compared with the experimental group.	Depression symptoms (yoga) (SMD 0.02; 95%CI 0.24 to 0.2; I^2^ UR; Tau^2^ = UR; k = 3; N = 314). Yoga may improve depression symptoms. - GRADE: moderate evidence (serious risk of bias).
Deprato et al., 2025 [8]	Objective original review: to summarize the evidence on effects of postpartum exercise on maternal postpartum depression outcomes and anxiety. N total of participants: 4.072. Population: postpartum women in their first year after childbirth *. Number of randomized clinical trials: 26. Outcome analyzed in this overview: severity of depression symptoms (not specified in inclusion criteria how they were assessed). Target intervention: treatment of depression symptoms. * Studies were included if most participants were in their first year postpartum.	Period of physical exercise: postnatal physical exercise. Physical exercise modalities analyzed in this overview: aerobic exercise, multimodal exercise, and yoga.	Multiple controls were analyzed in the meta-analysis: no intervention, education only, usual care for postpartum period, or minimal contact.	Depression symptoms post-intervention (aerobic exercise): (SMD −0.46; 95%CI −0.83 to −0.09; I^2^ = 82%; Tau^2^ = 0.28; k = 11; N = 857). Aerobic exercise improved depression symptoms post-intervention. - GRADE: moderate evidence (serious inconsistency). Depression symptoms post-intervention (yoga): (SMD −0.60; 95%CI −1.06 to −0.15; I^2^ = 0%; Tau^2^ = 0.00; k = 2; N = 81) Yoga improved depression symptoms post-intervention. - GRADE: moderate evidence (serious imprecision). Depression symptoms post-intervention (multimodal exercise): (SMD −1.16; 95%CI −2.3 to −0.01; I^2^ 95%; Tau^2^ = 1.21; k = 4; N = 320) Multimodal exercise improved depression symptoms post- intervention. - GRADE: moderate evidence (serious inconsistency). Changes in depression symptoms (follow-up)(aerobic exercise): (SMD −0.69; 95%CI −1.29 to −0.09; I^2^ = 92%; Tau^2^ = 0.78; k = 10; N = 735) Aerobic exercise improved changes in depression symptoms post-intervention to follow-up. GRADE: moderate evidence (serious inconsistency). Changes in depression symptoms (follow up)(multimodal exercise): (SMD −1.24; 95%CI −2.50 to 0.02; I^2^ 96%; Tau^2^ = 1.47; k = 4; N = 320) No difference between groups. - GRADE: low evidence (serious inconsistency, serious imprecision)
Ji et al., 2024 [20]	Objective original review: to summarize the evidence on the prevention and treatment effects of different physical exercise modalities on perinatal depression. N total of participants: 5.282. Population: pregnant women or women in labor. Number of randomized clinical trials: 48. Outcome analyzed in this overview: depression symptoms (any validated assessment scale) (e.g., EPDS, HAMA, CES-D). Target intervention: prevention and treatment of depression symptoms.	Period of physical exercise: Prenatal and postpartum physical exercise. Physical exercise modalities analyzed in this overview: aerobic exercise, yoga, movement in water, and multimodal exercise.	Multiple controls were analyzed in the meta-analysis: non-intervention, daily activities, routine care, standard care.	Prevention of depression symptoms (aerobic exercise): (SMD −0.27; 95%CI −0.49 to −0.05; I^2^ = UR; Tau^2^ = UR; k = 3; N = 829). Aerobic exercise prevented depression symptoms. Prevention of depression symptoms (yoga): (SMD −0.86; 95%CI −1.2 to −0.52; I^2^ = UR; Tau^2^ = UR; k = 4; N = 210). Yoga prevented depression symptoms. Prevention of depression symptoms (multimodal: aerobic + resistance): (SMD −0.24; 95%CI −0.5 to 0.02; I^2^ = UR; Tau^2^ = UR; k = 2; N = 230). No differences were found between groups. Treatment of depression symptoms (aerobic exercise): (SMD −0.89; 95%CI −1.40 to 0.38; I^2^ = UR; Tau^2^ = UR; k = 9; N = 922). No differences were found between groups. Treatment of depression symptoms (yoga): (SMD −0.74; 95%CI −1.15 to −0.32; I^2^ = UR; Tau^2^ = UR; k = 7; N = 442). Yoga improved depression symptoms. Treatment depression symptoms (movement in water) (SMD −0.75; 95%CI −1.62 to 0.11; I^2^ = UR; Tau^2^ = UR; k = 2; N = 400). No differences between groups. - GRADE: GRADE did not apply in subgroup analyses.
Pentland et al., 2022 [21]	Objective general review: to summarize the evidence on the impact of walking-based aerobic exercise on postpartum depression severity. N total of participants: 242. Population: postpartum women with a child up to 24 months of age with mild to moderate depression. Number of randomized clinical trials: 5. Outcome analyzed in this overview: depression symptoms (any validated assessment scale) (e.g., EPDS or Hamilton Depression Rating Scale) *. Target intervention: treatment of depression symptoms. * All studies used Edinburgh Postnatal Depression Scale.	Period of physical exercise: postpartum physical exercise. Physical exercise modalities analyzed in this overview: aerobic exercise (walking).	Multiple controls were analyzed in the meta-analysis: non-intervention, social support, and usual care.	Depression severity (aerobic exercise): (MD −4.01; 95%CI −7.18 to −0.84; I^2^ = 86%; Tau^2^ = 10.43; k = 5; N = 243). Walking improved depression symptoms from baseline to intervention end point. - GRADE: GRADE did not apply.
Pritchett et al., 2017 [22]	Objective general review: to summarize the evidence on the effectiveness of aerobic exercise on treatment and prevention of postpartum depressive symptoms. N total of participants: 1.734. Population: postpartum women (within <1 year postpartum). Number of randomized clinical trials: 13. Outcome analyzed in this overview: depressive symptoms (questionnaire ordiagnostic review)(e.g., EPDS or CES-D) *. Target intervention: treatment of depression symptoms. * Although one study used a clinical interview, we selected the meta-analyses focused exclusively on studies using Edinburgh Postnatal Depression Scale.	Period of physical exercise: postpartum physical exercise. Physical exercise modalities analyzed in this overview: aerobic exercise.	Multiple controls were analyzed in the meta-analysis: education or usual care.	Depression symptoms (aerobic exercise): (WMD −1.54; 95%CI −2.97 to −0.12; I^2^ =87%; Tau^2^ = UR; k = 10; N = 652). Aerobic exercise improved depression symptoms from baseline to intervention end point. - GRADE: GRADE did not apply.
Wang et al., 2024 [23]	Objective general review: to summarize the evidence to address and rank which exercise-based interventions are preferable to usual care/no intervention or another exercise intervention for postpartum depression. N total of participants: 1260. Population: women during pregnancy or postpartum period. Number of randomized clinical trials: 12. Outcome analyzed in this overview: depressive symptoms (EPDS, SDS and HDRS). Target intervention: treatment of depression symptoms.	Period of physical exercise: prenatal and/or postnatal physical exercise. Physical exercise modalities analyzed in this overview: pram walking, yoga, and multimodal exercise.	Multiple controls were analyzed in the meta-analysis: usual care or no therapy.	Depression symptoms (pram walking): (SMD −0.91; 95%CI −1.47 to −0.36; I^2^ = 49%; Tau^2^ = 0.15; k = 4 N = 155). Pram walking improved depression symptoms - GRADE: moderate evidence (serious risk of bias). Depression symptoms (yoga): (SMD −0.67; 95%CI −0.98 to −0.36; I^2^ = 4%; Tau^2^ = 0.00; k = 3 N = 175). Yoga improved depression symptoms. - GRADE: moderate evidence (serious risk of bias).
Xu et al., 2023 [24]	Objective general review: to summarize the evidence on the preventive and therapeutic effects of aerobic exercise for postpartum depression. N total of participants: 2867. Population: healthy pregnant women or postpartum depressive women (18 years old or higher). Number of randomized clinical trials: 26. Outcome analyzed in this overview: depressive symptoms (e.g., EPDS). Target intervention: prevention and treatment of depression symptoms.	Period of physical exercise: UR. Physical exercise modalities analyzed in this overview: aerobic exercise.	Multiple controls were analyzed in the meta-analysis: usual care.	Prevention + treatment (overall) Depression symptoms (aerobic exercise) (MD −1.90; 95%CI −2.58 to −1.21; I^2^ = 86%; Tau^2^ = UR; k = 26; N = 2.867) Aerobic exercise improved depression symptoms. Depression symptoms (prevention) (aerobic exercise): (MD −1.96; 95%CI −2.23 to −1.70; I^2^ = 84%; Tau^2^ = UR; k = 10 N = 1565) Aerobic exercise prevented depression symptoms. Depression symptoms (treatment) (aerobic exercise): (MD −1.04; 95%CI −1.78 to −0.30; I^2^ = 9%; Tau^2^ = UR; k = 10 N = 845). Aerobic exercise improved depression symptoms. - GRADE: GRADE did not apply.

Note: GRADE: Grading of Recommendations Assessment, Development and Evaluation; SMD: standardized mean difference; UR: unreported; MD: mean difference; g: Hedges’g; CI: confidence interval; k: number of articles analyzed; N: number of participants.

### 3.1. Methodological Quality: AMSTAR-2

Table 2 shows the methodological quality of the included reviews. The assessment of the potential impact of risk of bias in individual studies on the results of the meta-analysis (item 12) and the reporting of the funding sources of primary studies (item 10) were the items less checked. Only one out of seven reviews [7] explained the selection of the study design for inclusion (item 3) and included a justified list of excluded studies (item 7). Two out of seven reviews [8,22] completed a comprehensive search strategy (item 4) without methodological concerns. One out of seven reviews [21] described primary studies in sufficient detail (item 8). Three out of seven reviews [22,23,24] clearly explained having developed a prior protocol and reported any deviations from it (item 2). All studies except one [8] satisfactorily assessed the risk of bias in individual studies without methodological concerns. One out of seven reviews [24] did not account for risk of bias in individual studies during the interpretation or discussion of results. The percentage of agreement between ACM and JMS was 81.25%.

### 3.2. Overlap Between Meta-Analyses

**Figure 2:**
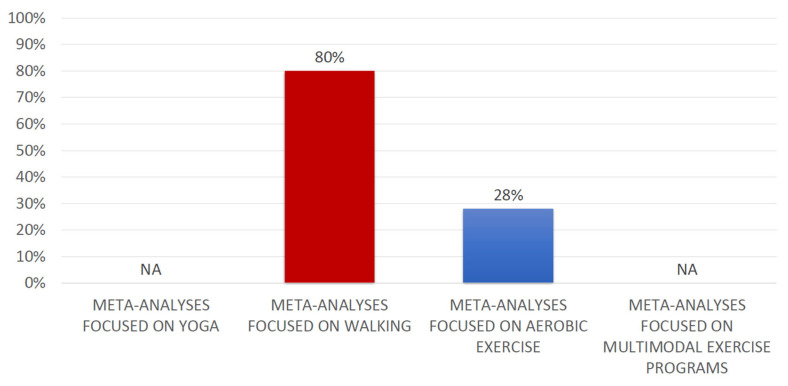
Degree of overlap for the different modalities of treatment. NA: More than one review evaluated this intervention, but it was not possible to know with certainty the studies meta-analyzed. Note: Movement in water was only analyzed by one review.

The degree of overlap between meta-analyses of interest was calculated for the following physical activity modalities: walking and aerobic exercises (Supplementary File S6). The degree of overlap between the meta-analyses evaluating both physical activity modalities was very high (walking CCA: 80%; aerobic exercise CCA: 28%). The degree of overlap for all analyses is depicted in Figure 2. We could not calculate the degree of overlap for meta-analyses evaluating yoga or multimodal exercise programs, and the reasons are reported in Supplementary File S6. In addition, no possibilities emerged to calculate the degree of overlap between meta-analyses considering movement in water since only one meta-analysis evaluated this intervention.

### 3.3. Effects of Movement in Water on Postpartum Depression Symptoms

One systematic review reported a meta-analysis evaluating the effects of movement in water on postpartum depression symptoms and found no differences between the experimental groups (movement in water) and control groups: SMD −0.75; 95%CI −1.62 to 0.11; I^2^ = UR; k = 2; N = 400; GRADE: UR [20].

### 3.4. Effects of Walking on Postpartum Depression Symptoms

Two systematic reviews reported meta-analyses evaluating the effects of walking on postpartum depression symptoms [21,23]. Wang et al. found the experimental groups (walking) may improve postpartum depression symptoms in comparison with control groups (SMD −0.91; 95%CI −1.47 to −0.36; I^2^ = 49%; k = 4; N = 155; GRADE: moderate evidence) [23]. Pentland et al. also found the experimental groups (walking) may improve postpartum depression symptoms specifically from baseline to the intervention end point when compared with the control groups (MD −4.01; 95%CI −7.18 to −0.84; I^2^ = 86%; k = 5; N = 243; GRADE: UR) [21].

### 3.5. Effects of Multimodal Exercise Programs on POSTPARTUM Depression Symptoms

Two systematic reviews reported meta-analyses evaluating the effects of multimodal exercise programs on postpartum depression symptoms and found different results considering the effects of these multimodal programs. Whereas Deprato et al. found the experimental groups (multimodal exercise programs) may be more effective than the control groups in improving postpartum depression symptoms at post-intervention (SMD −1.16; 95%CI −2.3 to −0.01; I^2^ = 95%; k = 4; N = 320; GRADE: moderate evidence) and follow-up (SMD −1.25; 95%CI −2.5 to −0.02; I^2^ 96%; k = 4; N = 320; GRADE: low evidence) [8], Ji et al. showed no differences between groups when specific time-points were not considered (SMD −0.24; 95%CI −0.5 to 0.02; I^2^ = UR; k = 2; N = 230; GRADE: UR) [20].

### 3.6. Effects of Aerobic Exercise on Postpartum Depression Symptoms

Four systematic reviews reported meta-analyses evaluating the effects of aerobic exercise on postpartum depression symptoms [8,20,22,24]. Xu et al. (MD −1.90; 95%CI −2.58 to −1.21; I^2^ = 86%; k = 26; N = 2.867; GRADE: UR) [24] found that the experimental groups (aerobic exercise) may be more effective than control groups in improving depression symptoms. Considering specific time-points, Deprato et al. found the experimental groups (aerobic exercise) may improve postpartum depression symptoms in comparison with the control groups at post-intervention (SMD −0.46; 95%CI −0.83 to −0.09; I^2^ = 82%; k = 11; N = 857; GRADE: moderate evidence) and post-intervention to follow-up (SMD −0.69; 95%CI −1.29 to −0.09; I^2^ = 92%; k = 10; N = 735; GRADE: moderate evidence) [8]. Pritchett et al. found positive results in favor of the experimental groups (aerobic exercise) in comparison with the control groups to improve postpartum depression symptoms from baseline to end of intervention (WMD −1.54; 95%CI −2.97 to −0.12; I^2^ = 87%; k = 10; N = 652; GRADE: UR) [22]. Finally, Ji et al. and Xu et al. included subgroups meta-analyses evaluating both treatment and prevention [20,24]. Whereas Xu et al. found positive results in favor of the experimental groups (aerobic exercise) in comparison with the control groups for preventing (MD −1.96; 95%CI −2.23 to −1.70; I^2^ 84%; k = 10; N = UR; GRADE: UR) and treating (MD −1.04; 95%CI −1.78 to −0.30; I^2^ 9%; k = 10; N = UR; GRADE: UR) postpartum depression [24], Ji et al. fund that the experimental groups (aerobic exercise) may be more effective than the control groups in the prevention of postpartum depression symptoms (SMD −0.27; 95%CI −0.49 to −0.05; I^2^ UR; k = 3; N = 829; GRADE: UR), but no differences between groups were found for the treatment of postpartum depression (SMD −0.89; 95%CI −1.40 to 0.38; I^2^ UR; k = 9; N = 922; GRADE: UR) [20].

### 3.7. Effects of Yoga on Postpartum Depression Symptoms

Four systematic reviews reported meta-analyses evaluating the effects of yoga on postpartum depression symptoms [7,8,20,23]. Davenport et al. (SMD 0.02; 95%CI 0.24 to 0.2; I^2^ = UR; k = 3; N = 314; GRADE: moderate evidence) [7] and Wang et al. (SMD −0.67; 95%CI −0.98 to −0.36; I^2^ = 4%; k = 3 N = 175; GRADE: moderate evidence) [23] found that the experimental groups (yoga) may be more effective than the control groups in improving depression symptoms. Considering specific time-points, Deprato et al. found the experimental groups (aerobic exercise) may be more effective in improving postpartum depression symptoms at post-intervention in comparison with the control groups (SMD −0.60; 95%CI −1.06 to −0.15; I^2^ = 0%; k = 2; N = 81; GRADE: moderate evidence) [8]. Finally, Ji et al. included subgroup meta-analyses evaluating both treatment and prevention and found positive results in favor of the experimental groups (yoga) in comparison with the control groups for preventing (SMD −0.86; 95%CI −1.2 to −0.52; I^2^ UR; k = 4; N = 210; GRADE: UR) and treating (SMD −0.74; 95%CI −1.15 to −0.32; I^2^ UR; k = 7; N = 442; GRADE: UR) postpartum depression symptoms [20].

## 4. Discussion

This overview of systematic reviews with meta-analysis aimed to synthesize the available evidence on the effectiveness of interventions focused on physical activity for improving depression symptoms in the postpartum period. After an exhaustive search of information, including eight e-databases, and a robust study selection process, a total of seven systematic reviews with meta-analyses were included in this overview. Overall, the included meta-analyses showed favorable results regarding the effects of physical activity-based interventions on postpartum depression symptoms. Considering specific physical activity modalities, the largest number of meta-analyses focused on aerobic exercise, yoga, or multimodal exercise programs. In all three cases, most meta-analyses found that aerobic exercise, yoga, and multimodal exercise programs could reduce postpartum depression symptoms. Furthermore, several meta-analyses explored the effectiveness of walking, finding positive results in favor of this intervention in comparison with the control groups in reducing postpartum depression symptoms. Finally, movement in water was only explored in one meta-analysis, and no differences were found between this intervention and control groups. Despite the positive results in favor of some interventions focused on physical activity in this field, it is important for readers, especially clinicians, to be aware of the following points, which are key to understanding whether the results found in this overview can be directly extrapolated to clinical practice.

### 4.1. Methodological Considerations, Clinical Implications, and Future Research

One of the main challenges in extrapolating clinical research into daily clinical practice is the lack of specific data on the type of physical activity being meta-analyzed. Although we tried to include only specific physical activity modalities that could provide the most accurate data for clinical practice, we believe there is still much to be done. For example, the included systematic reviews explored physical activity modalities such as aerobic exercise, yoga, movement in water, and multimodal exercise programs. These are all very broad concepts and can easily lead clinicians to ask questions such as the following: What style of yoga should I use? What does movement in water mean? Are these aquatic exercises focused on resistance or strength training, or both? What should we consider when we talk about aerobic exercise? All these questions remain open and should perhaps be resolved with subgroup analyses that will show results that are more applicable to clinical practice.

Another important point concerns whether the outcomes included in the evaluated meta-analyses focused on prevention or treatment. It is true that some systematic reviews included subgroups based on prevention and treatment [20,24], but most systematic reviews did not report in their objective whether they focused on prevention or treatment. Although we attempted to include the target of each intervention included, readers should be cautious with our report, as the authors of the systematic reviews did not clearly specify whether they focused on prevention or treatment. Differentiating outcomes for prevention and treatment is of vital importance, as it is not the same depending on whether women had depression at the beginning of the study or not. Furthermore, some authors have found differences in the effects of aerobic exercise between prevention and treatment [20]. Therefore, we encourage future systematic reviews to include subgroups based on prevention or treatment, or at least to specify in their objective whether they seek to prevent or treat symptoms of postpartum depression.

From a conceptual perspective, it is important to note that physical exercise, mind–body exercise, and regular physical activity are not the same. We have defined these three broad categories in our method, and furthermore, mind–body exercises have a working philosophy that is quite different from classic workouts focused on endurance, strength, or flexibility. Therefore, readers should be aware that some meta-analyses we found include interventions such as yoga under the umbrella of “aerobic exercise,” and this could have significantly increased the heterogeneity in meta-analyses and affected the interpretation of the results [24]. The same meta-analysis also included stretching within the overall concept of aerobic exercise [24], when it is likely that some researchers and clinicians did not include stretching exercises within the aerobic exercise category and instead included them in the flexibility exercise category. Another meta-analysis focused on studying walking, but some of the original studies focused on other exercise programs or evaluated walking as a modality inside of a multidisciplinary program [21]. Therefore, we encourage future systematic reviews to separate physical exercise from mind–body exercises, and even from regular physical activity, in their analyses to provide results that are more applicable to clinical practice.

Finally, it is also important to note that we have not found information about the importance of exploring prenatal depression status as a potential moderator of the effects of physical exercise, mind–body exercises, or regular physical exercise on postpartum depression. This information is needed in these meta-analyses since women who developed depression during pregnancy may be more likely to have postpartum depression and be less likely to perform physical exercise, contributing to biased results. Future research on this topic should explore this.

### 4.2. Limitations of This Overview

The results of this overview should not be directly extrapolated to the prevention or treatment of postpartum depression symptoms, as many meta-analyses did not specify whether they focused on one of these two factors. Furthermore, our results should not be extrapolated to people clinically diagnosed with depressive disorders, as the results are based on studies that assessed depression with self-report questionnaires.

## 5. Conclusions

Aerobic exercise, walking, yoga, and multimodal exercise programs may improve postpartum depression symptoms. Movement in water was not more effective than control groups for reducing this outcome. However, the results of our overview should be considered with caution, since important methodological and clinical implications have been discussed above and should guide the development of future systematic reviews on this topic.

## Figures and Tables

**Table 2:** The methodological quality of reviews (AMSTAR 2).

Author(s)	1	2	3	4	5	6	7	8	9	10	11	12	13	14	15	16
Davenport et al., 2018 [7]																
Deprato et al., 2025 [8]																
Ji et al., 2024 [20]																
Pentland et al., 2022 [21]																
Pritchett et al., 2017 [22]																
Wang et al., 2024 [23]																
Xu et al., 2023 [24]																

Note: Answers: red color: No; yellow color: Partially yes; green color: Yes. Items: AMSTAR 1: Did the research questions and inclusion criteria for the review include the components of PICO? AMSTAR 2: Did the report of the review contain an explicit statement that the review methods were established prior to the conduct of the review and did the report justify any significant deviations from the protocol? AMSTAR 3: Did the review authors explain their selection of the study designs for inclusion in the review? AMSTAR 4: Did the review authors use a comprehensive literature search strategy? AMSTAR 5: Did the review authors perform study selection in duplicate? AMSTAR 6: Did the review authors perform data extraction in duplicate? AMSTAR 7: Did the review authors provide a list of excluded studies and justify the exclusions? AMSTAR 8: Did the review authors describe the included studies in adequate detail? AMSTAR 9: Did the review authors use a satisfactory technique for assessing the risk of bias in individual studies that were included in the review? AMSTAR 10: Did the review authors report on the sources of funding for the studies included in the review? AMSTAR 11: If meta-analysis was performed did the review authors use appropriate methods for statistical combination of results? AMSTAR 12: If meta-analysis was performed, did the review authors assess the potential impact of bias in individual studies on the results of the meta-analysis or other evidence synthesis? AMSTAR 13: Did the review authors account for the risk of bias in individual studies when interpreting/discussing the results of the review? AMSTAR 14: Did the review authors provide a satisfactory explanation for, and discussion of, any heterogeneity observed in the results of the review? AMSTAR 15: If they performed quantitative synthesis did the review authors carry out an adequate investigation of publication bias (small study bias) and discuss its likely impact on the results of the review? AMSTAR 16: Did the review authors report any potential sources of conflict of interest, including any funding they received for conducting the review?

## Data Availability

No new data were created or analyzed in this study. Data sharing is not applicable to this article. All data relevant to the study are included in the article or are available as Supplementary Files.

## Supplementary Materials

The following supporting information can be downloaded at https://www.mdpi.com/article/10.3390/healthcare13121419/s1, Supplementary File S1: PRIOR checklist; Supplementary File S2: PRISMA 2020 abstract checklist; Supplementary File S3: Deviations Protocol; Supplementary File S4: Search strategies; Supplementary File S5: Full text analysis: included studies and excluded studies with reasons for exclusion; Supplementary File S6: Matrices of evidence and the corrected covered area (CCA) calculations considering the included meta-analyses.
