# Supplementary material for: What Interventions Focused on Physical Activity Could Improve Postpartum Depression Symptoms? An Overview of Systematic Reviews with Meta-Analysis
Source: Healthcare (Basel). 2025 Jun 13;13(12):1419. doi: 10.3390/healthcare13121419 (PMC12192973; doi:10.3390/healthcare13121419)
Supplement: Supplementary file 1 [file healthcare-13-01419-s001.zip › Suppl File 3 Deviations Protocol.pdf]

Supplementary File S3. Protocol deviations.

Objective

This overview eventually focused on depressive symptoms and diagnosis of depression was not considered.

Search strategies

The following e-databases were also searched: PsycINFO. This led us to develop a more comprehensive and robust search strategy.

Eligibility criteria

The following deviations were made to develop a more focused review aiming to send more direct clinical messages.

Population: Women with or without postpartum depression at baseline were considered.

I: Only meta-analysis focused on specific physical activity modalities were considered.

Outcomes: Only depression symptoms were considered.

Design: Only meta-analyses of randomized clinical trials were considered.

Data analysis

The results were only presented in the main text by type of physical activity modality.

Overlap

The degree of overlap was calculated when at least two two reviews meta-analyzed the same type of physical activity modality (e.g., yoga).
