# Supplementary material for: What Interventions Focused on Physical Activity Could Improve Postpartum Depression Symptoms? An Overview of Systematic Reviews with Meta-Analysis
Source: Healthcare (Basel). 2025 Jun 13;13(12):1419. doi: 10.3390/healthcare13121419 (PMC12192973; doi:10.3390/healthcare13121419)
Supplement: Supplementary file 1 [file healthcare-13-01419-s001.zip › Suppl File 4 Search strategies.pdf]

**Supplementary file S4.** Full search strategy for each e-database.

**CINAHL (date 19/02/2025)**

AB (exercise OR physical-activity OR training OR yoga OR yogic OR tai-chi OR taichi OR qigong OR qi-gong OR Tai-Ji OR Tai OR T'ai OR kung OR chung OR Baduanjin OR Wuqinxi OR Liuzijue OR Yijinjing OR qi-training OR gong OR taijiquan OR pilates OR walking OR sport*) AND AB (postpartum-depress*) AND AB (systematic OR meta-analysis OR metaanalysis OR meta-analyses OR metanalyses OR meta-review OR meta-analytic-review)

Search modes – Proximity.

**Studies retrieved = 29**

**Embase (date 19/02/2025)**

(exercise:ab,ti OR 'physical activity':ab,ti OR training:ab,ti OR yoga:ab,ti OR yogic:ab,ti OR 'tai chi':ab,ti OR taichi:ab,ti OR qigong:ab,ti OR 'qi gong':ab,ti OR 'tai ji':ab,ti OR tai:ab,ti OR t`ai:ab,ti OR kung:ab,ti OR chung:ab,ti OR baduanjin:ab,ti OR wuqinxi:ab,ti OR liuzijue:ab,ti OR yijinjing:ab,ti OR 'qi training':ab,ti OR gong:ab,ti OR taijiquan:ab,ti OR pilates:ab,ti OR walking:ab,ti OR sport*:ab,ti) AND ('postpartum depress*':ab,ti) AND (systematic:ab,ti OR 'meta analysis':ab,ti OR metaanalysis:ab,ti OR 'meta analyses':ab,ti OR metaanalyses:ab,ti OR 'meta review':ab,ti OR 'meta analytic review':ab,ti)

Search filter: conference abstracts were not considered.

**Studies retrieved = 46**

**Epistemonikos (date 19/02/2025)**

(title:((exercise OR physical-activity OR training OR yoga OR yogic OR tai-chi OR taichi OR qigong OR qi-gong OR Tai-Ji OR Tai OR T'ai OR kung OR chung OR Baduanjin OR Wuqinxi OR Liuzijue OR Yijinjing OR qi-training OR gong OR taijiquan OR pilates OR walking OR sport*)) OR abstract:((exercise OR physical-activity OR training OR yoga OR yogic OR tai-chi OR taichi OR qigong OR qi-gong OR Tai-Ji OR Tai OR T'ai OR kung OR chung OR Baduanjin OR Wuqinxi OR Liuzijue OR Yijinjing OR qi-training OR gong OR taijiquan OR pilates OR walking OR sport*))) AND (title:((postpartum)) OR abstract:((postpartum))) AND (title:((systematic OR meta-analysis OR metaanalysis OR meta-analyses OR metanalyses OR meta-review OR meta-analytic-review)) OR abstract:((systematic OR meta-analysis OR metaanalysis OR meta-analyses OR metanalyses OR meta-review OR meta-analytic-review)))

**Studies retrieved = 215**

**PsycINFO (date 19/02/2025)**

abstract((exercise OR physical-activity OR training OR yoga OR yogic OR tai-chi OR taichi OR qigong OR qi-gong OR Tai-Ji OR Tai OR T'ai OR kung OR chung OR Baduanjin OR Wuqinxi OR Liuzijue OR Yijinjing OR qi-training OR gong OR taijiquan OR pilates OR walking OR sport*) ) AND abstract((postpartum-depress*) ) AND abstract((systematic OR meta-analysis OR metaanalysis OR meta-analyses OR metanalyses OR meta-review OR meta-analytic-review) )

Search filter: theses and dissertations were not considered.

**Studies retrieved = 18**

**PubMed (date 19/02/2025)**

(exercise [tiab] OR physical-activity [tiab] OR training [tiab] OR yoga [tiab] OR yogic [tiab] OR tai-chi [tiab] OR taichi [tiab] OR qigong [tiab] OR qi-gong [tiab] OR Tai-Ji [tiab] OR Tai [tiab] OR T'ai [tiab] OR kung [tiab] OR chung [tiab] OR Baduanjin [tiab] OR Wuqinxi [tiab] OR Liuzijue [tiab] OR Yijinjing [tiab] OR qi-training [tiab] OR gong [tiab] OR taijiquan [tiab] OR pilates [tiab] OR walking [tiab] OR sport* [tiab]) AND (postpartum-depress* [tiab]) AND (systematic [tiab] OR meta-analysis [tiab] OR metaanalysis [tiab] OR meta-analyses [tiab] OR metaanalyses [tiab] OR meta-review [tiab] OR meta-analytic-review [tiab])

**Studies retrieved = 54**

**Scopus (date 19/02/2025)**

TITLE-ABS-KEY (exercise OR physical-activity OR training OR yoga OR yogic OR tai-chi OR taichi OR qigong OR qi-gong OR Tai-Ji OR Tai OR T'ai OR kung OR chung OR Baduanjin OR Wuqinxi OR Liuzijue OR Yijinjing OR qi-training OR gong OR taijiquan OR pilates OR walking OR sport*) AND TITLE-ABS-KEY (postpartum-depress*) AND TITLE-ABS-KEY (systematic OR meta-analysis OR metaanalysis OR meta-analyses OR metanalyses OR meta-review OR meta-analytic-review)

**Studies retrieved = 81**

**SPORTDiscus (date 19/02/2025)**

AB (exercise OR physical-activity OR training OR yoga OR yogic OR tai-chi OR taichi OR qigong OR qi-gong OR Tai-Ji OR Tai OR T'ai OR kung OR chung OR Baduanjin OR Wuqinxi OR Liuzijue OR Yijinjing OR qi-training OR gong OR taijiquan OR pilates OR walking OR sport*)

AND AB (postpartum-depress*) AND AB (systematic OR meta-analysis OR metaanalysis OR meta-analyses OR metanalyses OR meta-review OR meta-analytic-review)

Search modes – Proximity.

**Studies retrieved = 4**

**The Cochrane Library (date 19/02/2025)**

| ID | Search | Results |
|---|---|---|
| #1 | (postpartum):ti,ab,kw | 14972 |
| #2 | (depression):ti,ab,kw | 109634 |
| #3 | (depressive):ti,ab,kw | 38853 |
| #4 | (#1) AND (#2 OR #3) | 3112 |

Search filters: Cochrane reviews and Cochrane protocols.

**Studies retrieved = 45**
