# Supplementary material for: What Interventions Focused on Physical Activity Could Improve Postpartum Depression Symptoms? An Overview of Systematic Reviews with Meta-Analysis
Source: Healthcare (Basel). 2025 Jun 13;13(12):1419. doi: 10.3390/healthcare13121419 (PMC12192973; doi:10.3390/healthcare13121419)
Supplement: Supplementary file 1 [file healthcare-13-01419-s001.zip › Suppl File 5 List studies full analysis.pdf]

Supplementary file S5. Full text analysis: included studies and excluded studies with reasons for exclusion.

Note: Under the following table is reported the table with the analysis of studies at full text that were found during manual searching.

Included (k=8)

Language restriction (k=2)

No full text available (k=1)

No intervention of interest (k=1)

No meta-analysis (k=26)

No meta-analysis of interest (k=11)

No outcome of interest (k=1)

No research design of interest (k=9)

Preprint (k=1)

Study protocol (k=4)

Study	Reason
1. Cordero, M.A.; López, A.S.; Blanque, R.R.; Segovia, J.N.; Cano, M.P.; López-Contreras, G.; Villar, N.M. Physical activity by pregnant women and its influence on maternal and foetal parameters; a systematic review. *Nutr. Hosp.* **2014**, *30*, 719–726.	No meta-analysis.
2. Alderdice, F.; McNeill, J.; Lynn, F. A systematic review of systematic reviews of interventions to improve maternal mental health and well-being. *Midwifery* **2013**, *29*, 389–399.	No research design of interest.
3. Austin, M.P.V.; Middleton, P.; Reilly, N.M.; Highet, N.J. Detection and management of mood disorders in the maternity setting: The australian clinical practice guidelines. *Women Birth* **2013**, *26*, 2–9.	No meta-analysis.
4. Baskar, T.; Mahajan, N.M.; Gaikwad, J. Interventions For Postpartum Depression: Systematic Review. *NeuroQuantology* **2022**, *20*, 1136–1146	No meta-analysis.
5. Brown, A.M.; Robinson, A.; Jones, F.; Smith, A.; Williams, P.; Hanley, J. The effectiveness of prescription exercises for women diagnosed with postnatal depression: A systematic review. *MIDIRS Midwifery Dig.* **2017**, *27*, 488–495.	No meta-analysis.
6. Brown, J.; Alwan, N.A.; West, J.; Brown, S.; McKinlay, C.J.; Farrar, D.; Crowther, C.A. Lifestyle interventions for the treatment of women	No meta-analysis of interest.

	with gestational diabetes. *Cochrane Database Syst. Rev.* **2017**, CD011970. https://doi.org/10.1002/14651858.CD011970.pub2	Note: Postnatal depression was only analyzed by one study.
7.	Levitt, C.; Shaw, E.; Wong, S.; Kaczorowski, J.; Springate, R.; Sellors, J.; Enkin, M. Systematic review of the literature on postpartum care: Methodology and literature search results. *Birth* **2004**, *31*, 196–202.	No meta-analysis.
8.	Carter, T.; Bastounis, A.; Guo, B.; Jane Morrell, C. The effectiveness of exercise-based interventions for preventing or treating postpartum depression: A systematic review and meta-analysis. *Arch. Women's Ment. Health* **2019**, *22*, 37–53	No meta-analysis of interest.<br>Note: No meta-analysis by physical exercise modalities (e.g., yoga, aerobic exercise, resistance exercises...)
9.	Chow, R.; Huang, E.; Li, A.; Li, S.; Fu, S.Y.; Son, J.S.; Foster, W.G. Appraisal of systematic reviews on interventions for postpartum depression: Systematic review. *BMC Pregnancy Childbirth* **2021**, *21*, 18	No research design of interest.
10.	Craig, M.; Howard, L. Postnatal depression. *BMJ Clin. Evid.* **2009**, *2009*, 1407.	No research design of interest.
11.	Daley, A.J.; MacArthur, C.; Winter, H. The Role of Exercise in Treating Postpartum Depression: A Review of the Literature. *J. Midwifery Women's Health* **2007**, *52*, 56–62.	No meta-analysis.
12.	Davenport, M.H.; McCurdy, A.P.; Mottola, M.F.; Skow, R.J.; Meah, V.L.; Poitras, V.J.; Garcia, A.J.; Gray, C.E.; Barrowman, N.; Riske, L.; et al. Impact of prenatal exercise on both prenatal and postnatal anxiety and depressive symptoms: A systematic review and meta-analysis. *Br. J. Sports Med.* **2018**, *52*, 1376–1385	Included.
13.	Deligiannidis, K.M.; Freeman, M.P. Complementary and alternative medicine therapies for perinatal depression. *Best Pract. Res. Clin. Obstet. Gynaecol.* **2014**, *28*, 85–95.	No meta-analysis.
14.	Dennis, C.L.; Brown, H.K.; Morrell, J. Interventions (other than psychosocial, psychological and pharmacological) for preventing postpartum depression. *Cochrane Database Syst. Rev.* **2016**, *2016*, CD012201.	Study protocol.
15.	Dennis, C.L.; Brown, J.V.E.; Brown, H.K. Interventions (other than psychosocial, psychological and pharmacological) for treating	Study protocol.

postpartum depression. *Cochrane Database Syst. Rev.* **2019**, *2019*, CD013460	
16. Deprato, A.; Ruchat, S.-M.; Ali, M.U.; Cai, C.; Forte, M.; Gierc, M.; Meyer, S.; Sjwed, T.N.; Shirazi, S.; Matenchuk, B.A.; et al. Impact of postpartum physical activity on maternal depression and anxiety: A systematic review and meta-analysis. *Br. J. Sports Med.* **2025**, *59*, 550–561. https://doi.org/10.1136/bjsports-2024-108478.	Included.
17. DiPietro, L.; Evenson, K.R.; Bloodgood, B.; Sprow, K.; Troiano, R.P.; Piercy, K.L.; Vaux-Bjerke, A.; Powell, K.E. Benefits of Physical Activity during Pregnancy and Postpartum: An Umbrella Review. *Med. Sci. Sports Exerc.* **2019**, *51*, 1292–1302.	No research design of interest.
18. Evenson, K.R.; Brown, W.J.; Brinson, A.K.; Budzynski-Seymour, E.; Hayman, M. A review of public health guidelines for postpartum physical activity and sedentary behavior from around the world. *J. Sport Health Sci.* **2024**, *13*, 472–483.	No meta-analysis.
19. Field, T. Yoga research review. *Complement. Ther. Clin. Pract.* **2016**, *24*, 145–161.	No meta-analysis.
20. Gunabalasingam, S.; Kyrka, A.; Hopkins, L.; Lebrett, R.; Dyer, E.; Forde, R.; Heslehurst, N.; Meek, C.L.; Schoenaker, D.A.J.M.; Flynn, A.C.; et al. Interventions in women with type 2 diabetes mellitus in the pre-pregnancy, pregnancy and postpartum periods to optimise care and health outcomes: A systematic review. *Diabet. Med.* **2024**, *42*, e15474.	No meta-analysis.
21. He, L.; Soh, K.L.; Huang, F.; Khaza'ai, H.; Geok, S.K.; Vorasiha, P.; Chen, A.; Ma, J. The impact of physical activity intervention on perinatal depression: A systematic review and meta-analysis. *J. Affect. Disord.* **2023**, *321*, 304–319	No meta-analysis of interest.<br>Note: No specific meta-analyses focused on randomized clinical trials were performed.
22. Hicks, L.E.; Graf, M.D.; Yeo, S. Prenatal exercise and its effects on postpartum mental health: Systematic review and meta-analysis. *Arch. Women's Ment. Health* **2025**, *28*, 515–524.	No meta-analysis of interest.<br>Note: No meta-analysis by physical exercise modalities (e.g., yoga, aerobic exercise, resistance exercises...)

23. Ji, M.; Li, R.; Xu, Y. Meta-analysis of the effect of different exercise modalities in the prevention and treatment of perinatal depression. *J. Affect. Disord.* **2024**, *350*, 442–451	Included.
24. Kimber, M.L.; Meyer, S.; McHugh, T.L.; Thornton, J.; Khurana, R.; Sivak, A.; Davenport, M.H. Health Outcomes after Pregnancy in Elite Athletes: A Systematic Review and Meta-analysis. *Med. Sci. Sports Exerc.* **2021**, *53*, 1739–1747. https://doi.org/10.1249/MSS.0000000000002617.	No meta-analysis of interest.
25. Kołomańska-Bogucka, D.; Mazur-Bialy, A.I. Physical activity and the occurrence of postnatal depression—A systematic review. *Medicina* **2019**, *55*, 560).	No meta-analysis.
26. Kwon, R.; Kasper, K.; London, S.; Haas, D.M. A systematic review: The effects of yoga on pregnancy. *Eur. J. Obstet. Gynecol. Reprod. Biol.* **2020**, *250*, 171–177.	No meta-analysis.
27. Lima-De-La-Iglesia C, Magni E, Botello-Hermosa A, Guerra-Martín MD. Benefits of Complementary Therapies During Pregnancy, Childbirth and Postpartum Period: A Systematic Review. Healthcare (Basel, Switzerland). 2024;12(23).	No meta-analysis.
28. Lima-De-La-Iglesia, C.; Magni, E.; Botello-Hermosa, A.; Guerra-Martín, M.D. Benefits of Complementary Therapies During Pregnancy, Childbirth and Postpartum Period: A Systematic Review. *Healthcare* **2024**, *12*, 2481	No outcome of interest.<br>Note: Depression symptoms was not considered as outcome measure.
29. Lu, X.; Yang, Z.; Liu, F.; Zhou, Y.; Xu, Y.; Zhuo, Y.; et al. Effectiveness of non-pharmacological treatments for postpartum depression: An umbrella review protocol. *BMJ Open* **2023**, *13*, e066395).	Study protocol.
30. Marconcin, P.; Peralta, M.; Gouveia, É.R.; Ferrari, G.; Carraça, E.; Ihle, A.; Marques, A. Effects of exercise during pregnancy on postpartum depression: A systematic review of meta-analyses. *Biology* **2021**, *10*, 1331	No research design of interest.
31. McCloskey, R.J.; Reno, R. Complementary health approaches for postpartum depression: A systematic review. *Soc. Work. Ment. Health* **2019**, *17*, 106–128	No meta-analysis.
32. McCurdy, A.P.; Boulé, N.G.; Sivak, A.; Davenport, M.H. Effects of Exercise on Mild-to-Moderate Depressive Symptoms in the	No meta-analysis of interest.

Postpartum Period: A Meta-analysis. *Obstet. Gynecol.* **2017**, *129*, 1087–1097.	Note: No meta-analysis by physical exercise modalities (e.g., yoga, aerobic exercise, resistance exercises...)
33. Mottola, M.F.; Davenport, M.H.; Ruchat, S.M.; Davies, G.A.; Poitras, V.; Gray, C.; Garcia, A.J.; Barrowman, N.; Adamo, K.B.; Duggan, M.; et al. No. 367-2019 Canadian Guideline for Physical Activity throughout Pregnancy. *J. Obstet. Gynaecol. Can.* **2018**, *40*, 1528–1537.	No full text available.<br>Note: We did not find information on the website of the journal about how to request information to the corresponding author.
34. Moufang Ji Li, R.; Hu, T.; Chen, J. Effects of different non-pharmacological interventions on postpartum depression in pregnant women: A network Meta-analysis. *Chin. J. Nurs.* **2024**, *59*, 228–235.	Language restriction.<br>Note: This article was published in other language rather than English or Spanish.
35. Munns, L.; Spark, N.; Crossland, A.; Preston, C. The effects of yoga-based interventions on postnatal mental health and well-being: A systematic review. *Heliyon* **2024**, *10*, e25455.	No meta-analysis.
36. Nakamura, A.; van der Waerden, J.; Melchior, M.; Bolze, C.; El-Khoury, F.; Pryor, L. Physical activity during pregnancy and postpartum depression: Systematic review and meta-analysis. *J. Affect. Disord.* **2019**, *246*, 29–41.	No meta-analysis of interest.<br>Note: No meta-analysis by physical exercise modalities (e.g., yoga, aerobic exercise, resistance exercises...)
37. Nascimento, S.L.; Surita, F.G.; Cecatti, J.G. Physical exercise during pregnancy: A systematic review. *Curr. Opin. Obstet. Gynecol.* **2012**, *24*, 387–394..	No meta-analysis.
38. Nillni, Y.I.; Mehralizade, A.; Mayer, L.; Milanovic, S. Treatment of depression, anxiety, and trauma-related disorders during the perinatal period: A systematic review. *Clin. Psychol. Rev.* **2018**, *66*, 136–148.	No meta-analysis.
39. Nnate, D.A.; Ajayi, K.V.; Hossain, M.M.; Guerby, P. Effectiveness of psychosocial interventions for hypertensive disorders in pregnancy: A systematic review and meta-analysis. *medRxiv* **2022**, https://doi.org/10.1101/2022.01.13.22269011.	Preprint.

40. Norhamazi, I.; Linoby, A.; Abd Latif, R.; Mursyid, S.; Naimah, M.; Haslan, A.N.; Noor, M.A.; Zamri, F.N. Exercise intervention up to 6 months postpartum improves the outcome of Edinburgh postnatal depression scale. *J. Phys. Educ. Sport* **2022**, *22*, 2127–2133.	No meta-analysis of interest.<br>Note: No meta-analysis by physical exercise modalities (e.g., yoga, aerobic exercise, resistance exercises...)
41. O'Connor, E.; Senger, C.A.; Henninger, M.; Gaynes, B.N.; Coppola, E.; Soulsby Weyrich, M. *Interventions to Prevent Perinatal Depression: Evidence Report and Systematic Review for the US Preventive Services Task Force*; American Medical Association: Chicago, IL, USA, 2019. https://doi.org/10.1001/jama.2018.20865	No research design of interest.<br>Note: This article was not published in a peer-reviewed journal.
42. Okamoto, R.; Terasawa, E.; Usui, A.; Matsushima, M.; Okayama, H. The effects of online facial muscle training with resonance vocalization on mental health in postpartum women: A single-arm pilot study. *Women's Health* **2024**, *20*, 17455057241286201.	No meta-analysis.
43. Osumi, A.; Kanejima, Y.; Ishihara, K.; Ikezawa, N.; Yoshihara, R.; Kitamura, M.; Izawa, K.P. Effects of Sedentary Behavior on the Complications Experienced by Pregnant Women: A Systematic Review. *Reprod. Sci.* **2024**, *31*, 352–365..	No meta-analysis.
44. Paz Fernández, N.; González González, Y.; Alonso Calvete, A.; Da Cuña Carrera, I. Effects of exercise in pregnant and postpartum depression—A systematic review. *Clin. Investig. Ginecol. Obstetricia* **2021**, *48*, 100683	No meta-analysis.
45. Pentland, V.; Spilsbury, S.; Biswas, A.; Mottola, M.F.; Paplinskie, S.; Mitchell, M.S. Does walking reduce postpartum depressive symptoms? A systematic review and meta-analysis of randomized controlled trials. *J. Women's Health* **2022**, *31*, 555–563	Included.
46. Poyatos-León, R.; García-Hermoso, A.; Sanabria-Martínez, G.; Álvarez-Bueno, C.; Cavero-Redondo, I.; Martínez-Vizcaíno, V. Effects of exercise-based interventions on postpartum depression: A meta-analysis of randomized controlled trials. *Birth* **2017**, *44*, 200–208.	No meta-analysis of interest.<br>Note: No meta-analysis by physical exercise modalities (e.g., yoga, aerobic exercise, resistance exercises...)

47. Pritchett, R.V.; Daley, A.J.; Jolly, K. Does aerobic exercise reduce postpartum depressive symptoms? A systematic review and meta-analysis. *Br. J. Gen. Pract.* **2017**, *67*, e684–e691	Included.
48. Qi, W.; Huang, S.; Zhao, J.; Cui, X.; Wei, Z.; Cui, G.; Guo, Q.; Hu, J. The preventive effect of psychological and psychosocial interventions on postpartum depression: An overview of systematic reviews. *J. Psychiatr. Res.* **2025**, *182*, 21–33.	No research design of interest.
49. Qian, J.; Sun, S.; Liu, L.; Yu, X. Effectiveness of nonpharmacological interventions for reducing postpartum fatigue: A meta-analysis. *BMC Pregnancy Childbirth* **2021**, *21*, 622.	No meta-analysis of interest.<br>Note: No meta-analysis by physical exercise modalities (e.g., yoga, aerobic exercise, resistance exercises...)
50. Christian, S.J. Meta-Analysis of the Effectiveness of Biological and Non-Biological Treatments for Postpartum Depression. Ph.D. Thesis, Brigham Young University, Provo, UT, USA, 2013..	No meta-analysis.
51. Sarah Christian. Meta-analysis of the effectiveness of biological and non-biological treatments for postpartum depression. Dissertation Abstracts International: Section B: The Sciences and Engineering [Internet]. 2013; Disponible en: https://www.epistemonikos.org/en/documents/bb0a9b9406a03b5906fd1d07dc6f2b770d24f032	No research design of interest.<br>Note: Dissertation.
52. Sha, L.; Zhang, A.; Fan, X.; Cong, S.; Wang, R.; Sun, X.; et al. Evidence summary of non-pharmacological interventions for maternal postpartum depression. *Chin. J. Nurs.* **2022**, *57*, 2977–2984.	No research design of interest.
53. Shaw, E.; Kaczorowski, J. Postpartum Care—What's New? *Curr. Opin. Obstet. Gynecol.* **2007**, *19*, 561–567	No meta-analysis.
54. Sousa Pérez, A.; Soto González, M. Efectos del ejercicio acuático en el embarazo, una revisión sistemática. *Fisioterapia* **2022**, *44*, 51–61.	No meta-analysis.
55. Szuhany, K.L.; Sullivan, A.J.; Gills, J.L.; Kredlow, M.A. The impact of exercise interventions on sleep in adult populations with depression, anxiety, or posttraumatic stress: Review of the current evidence and future directions. *J. Behav. Med.* **2025**, *48*, 4–21. https://doi.org/10.1007/s10865-024-00532-z.	No meta-analysis.

56. Tian, H.; Han, S.; Hu, J.; Peng, X.; Zhang, W.; Wang, W.; Qi, X.; Teng, J. The effectiveness of Tai Chi for postpartum depression: A protocol for systematic review and meta-analysis. *Medicine* **2021**, *100*, e28176. https://doi.org/10.1097/MD.0000000000028176..	Study protocol.
57. Turner, J.; Clanchy, K.; Vincze, L. Telehealth interventions for physical activity and exercise participation in postpartum women: A quantitative systematic review. *Prev. Med.* **2023**, *167*, 107413. https://doi.org/10.1016/j.ypmed.2022.107413.	No meta-analysis.
58. Wang, J.; Carru, C.; Sedda, S.; Fiori, P.L.; Li, Z.; Chen, Z. Comparative impact of exercise-based interventions for postpartum depression: A Bayesian network meta-analysis. *Int. J. Gynecol. Obstet.* **2024**, *165*, 67–75.	Included.
59. Xie, H.; Cong, S.; Wang, R.; Sun, X.; Han, J.; Ni, S.; Zhang, A. Effect of eHealth interventions on perinatal depression: A meta-analysis. *J. Affect. Disord.* **2024**, *354*, 160–172.	No intervention of interest.<br>Note: No specific meta-analysis was performed considering exercise, regular physical activity, or mind-body exercises.
60. Xu, H.; Liu, R.; Wang, X.; Yang, J. Effectiveness of aerobic exercise in the prevention and treatment of postpartum depression: Meta-analysis and network meta-analysis. *PLoS ONE* **2023**, *18*, e0287650	Included.
61. Yahya, N.F.S.; Teng, N.I.M.F.; Das, S.; Juliana, N. Nutrition and physical activity interventions to ameliorate postpartum depression: A scoping review. *Asia Pac. J. Clin. Nutr.* **2021**, *30*, 662–674.	No meta-analysis.
62. Yu, H.; Mu, Q.; Lv, X.; Chen, S.; He, H. Effects of an exercise intervention on maternal depression, anxiety, and fatigue: A systematic review and meta-analysis. *Front. Psychol.* **2024**, *15*, 1473710	No meta-analyses of interest.<br>Note: no subgroups by exercise modalities focused on postpartum depression were performed.
63. Yuan, M.; Chen, H.; Chen, D.; Wan, D.; Luo, F.; Zhang, C.; Nan, Y.; Bi, X.; Liang, J. Effect of physical activity on prevention of postpartum depression: A dose-response meta-analysis of 186,412 women. *Front. Psychiatry* **2022**, *13*, 984677.	No meta-analysis of interest.<br>Note: Although this review performed subgroup by type of physical activity (work, sports, and household), no specific analysis by exercise

	modalities were included. In fact, the authors included different exercise modalities such as yoga, pilates, and walking under the global term sports.
64. Jiang, X.; Tan, Z.; Nie, Y. A Meta-analysis of Effect of Exercise on Postpartum Depression Symptoms. *J. Wuhan Inst. Phys. Educ.* **2021**, *55*, 71–78.	Language restriction.<br><br>Note: No English or Spanish study.

Manual search

Study	Reason	Study where was found
Lumley, J.; Austin, M.-P.; Mitchell, C. Intervening to reduce depression after birth: a systematic review of the randomized trials. *Int. J. Technol Assess Health Care.* **2004,** 20(2), 128–44. https://doi.org/10.1017/s0266462304000911<br><br>.	No intervention of interest.	Alderdice, F.; McNeill, J.; Lynn, F. A systematic review of systematic reviews of interventions to improve maternal mental health and well-being. *Midwifery* **2013**, *29*, 389–399.<br>.
Bledsoe, S. E., & Grote, N. K. Treating Depression During Pregnancy and the Postpartum: A Preliminary Meta-Analysis. *Research on Social Work Practice*, **2006**, 16(2), 109-120. https://doi.org/10.1177/1049731505282202<br><br>.	No intervention of interest.	Alderdice, F.; McNeill, J.; Lynn, F. A systematic review of systematic reviews of interventions to improve maternal mental health and well-being. *Midwifery* **2013**, *29*, 389–399.<br>.
Daley, A.J.; MacArthur, C.; Winter, H. The Role of Exercise in Treating Postpartum Depression: A Review of the Literature. *J. Midwifery Women's Health* **2007**, *52*, 56–62.	No meta-analysis of interest.<br><br>Note: Subgroup meta-analyses focused on randomized clinical trials were not performed.	Chow, R.; Huang, E.; Li, A.; Li, S.; Fu, S.Y.; Son, J.S.; Foster, W.G. Appraisal of systematic reviews on interventions for postpartum depression: Systematic review.

		BMC Pregnancy Childbirth **2021**, *21*, 18
Mottola, M.F.; Davenport, M.H.; Ruchat, S.M.; Davies, G.A.; Poitras, V.; Gray, C.; Garcia, A.J.; Barrowman, N.; Adamo, K.B.; Duggan, M.; et al. No. 367-2019 Canadian Guideline for Physical Activity throughout Pregnancy. *J. Obstet. Gynaecol. Can.* **2018**, *40*, 1528–1537.	No meta-analysis.	Mottola, M.F.; Davenport, M.H.; Ruchat, S.M.; Davies, G.A.; Poitras, V.; Gray, C.; Garcia, A.J.; Barrowman, N.; Adamo, K.B.; Duggan, M.; et al. No. 367-2019 Canadian Guideline for Physical Activity throughout Pregnancy. *J. Obstet. Gynaecol. Can.* **2018**, *40*, 1528–1537.
O'Connor, E.; Senger, C.A.; Henninger, M.; Gaynes, B.N.; Coppola, E.; Soulsby Weyrich, M. *Interventions to Prevent Perinatal Depression: Evidence Report and Systematic Review for the US Preventive Services Task Force*; American Medical Association: Chicago, IL, USA, 2019. https://doi.org/10.1001/jama.2018.20865.	No meta-analysis of interest.<br><br>Note: Physical activity interventions were not specifically meta-analyzed.	O'Connor, E.; Senger, C.A.; Henninger, M.; Gaynes, B.N.; Coppola, E.; Soulsby Weyrich, M. *Interventions to Prevent Perinatal Depression: Evidence Report and Systematic Review for the US Preventive Services Task Force*; American Medical Association: Chicago, IL, USA, 2019. https://doi.org/10.1001/jama.2018.20865
