# Supplementary material for: What Interventions Focused on Physical Activity Could Improve Postpartum Depression Symptoms? An Overview of Systematic Reviews with Meta-Analysis
Source: Healthcare (Basel). 2025 Jun 13;13(12):1419. doi: 10.3390/healthcare13121419 (PMC12192973; doi:10.3390/healthcare13121419)
Supplement: Supplementary file 1 [file healthcare-13-01419-s001.zip › Suppl File 6 Overlap.pdf]

Supplementary file S6. Matrices of evidence and the corrected covered area (CCA) calculations considering the included meta-analyses. META-ANALYSES FOCUSED ON YOGA

Note: The following reviews were not included in the overlap calculation:

- Davenport et al. 2018 (the authors did not specify the studies that were meta-analyzed)
- Deprato et al. 2025 (the authors did not include the references that were meta-analyzed in the list of references)
- Ji et al. 2024 (the authors did not specify the studies that were meta-analyzed)

THE DEGREE OF OVERLAP COULD NOT BE CALCULTAED DUE TO ONLY ONE SYSTEMATIC REVIEW REPORTED THE STUDIES THAT WERE META-ANALYZED IN THE META-ANALYSES OF INTEREST (SEE BELOW)

**Number of studies without accounting for duplicates**	**Primary research (references)**	**Systematic reviews where are included primary research**
1.	Buttner, M.M.; Brock, R.L.; O'Hara, M.W.; Stuart, S. Efficacy of yoga for depressed postpartum women: A randomized controlled trial. *Complement. Ther. Clin. Pract.* **2015**, *21*, 94–100. https://doi.org/10.1016/j.ctcp.2015.03.003	1. Wang et al. 2024
2.	Reyad, M., El Refaye, G., Awad, M., Gabr. A.. Effect of yoga on postpartum depression: a randomized controlled trial."*Egyptian Journal of Physical Therapy*, **2022**, *1*, 21-27.	2. Wang et al. 2024
3.	Shu, L., Tan, C., Wu, C., Xi, M., Tan, H., Zhao, W., Qu, Q**.** Effects of Mindfulness-based Yoga Group Intervention on Maternal Postpartum Depression Following the Second Childbirth. Chinese General Practice J., **2019,** *22*, 2739-2743. https://doi.org/10.12114/j.issn.1007-9572.2019.00.354.	3. Wang et al. 2024

META-ANALYSES FOCUSED ON WALKING

$$CCA = \frac{N-r}{rc-r} = \frac{9-5}{10-5} \quad \frac{4}{5} = 0.8 = 80\%$$

Note: N is the total number of original studies (including duplicates) in the meta-analyses of interest (the sum of all checked boxes in the citation matrix). Furthermore, r is the number of original studies without accounting for duplicates. Finally, c is the number of systematic reviews included in the evidence matrix (k=2). CCA = corrected covered area.

**Number of studies without accounting for duplicates**	**Primary research (references)**	**Systematic reviews where are included primary research**
1.	Armstrong, K.; Edwards, H. The effects of exercise and social support on mothers reporting depressive symptoms: A pilot randomized controlled trial. *Int. J. Ment. Health Nurs.* **2003**, *12*, 130–138	1. Pentland et al. 2022<br>2. Wang et al. 2024

2.	Armstrong, K.; Edwards, H. The effectiveness of a pram-walking exercise programme in reducing depressive symptomatology for postnatal women. *Int. J. Nurs. Pract.* **2004**, *10*, 177–194. https://doi.org/10.1111/j.1440-172X.2004.00478.x.	3. Pentland et al. 2022<br>4. Wang et al. 2024
3.	Da Costa, D.; Lowensteyn, I.; Abrahamowicz, M.; Ionescu-Ittu, R.; Dritsa, M.; Rippen, N.; Cervantes, P.; Khalifé, S. A randomized clinical trial of exercise to alleviate postpartum depressed mood. *J. Psychosom. Obstet. Gynecol.* **2009**, *30*, 191–200.	5. Pentland et al. 2022
4.	Daley, A.J.; Winter, H.; Grimmett, C.; McGuinness, M.; McManus, R.; MacArthur, C. Feasib-ility of an exercise intervention for women with postnatal depression: A pilot randomised controlled trial. *Br. J. Gen. Pract.* **2008**, *58*, 178–183. https://doi.org/10.3399/bjgp08x277195.	6. Pentland et al. 2022<br>7. Wang et al. 2024
5.	Forsyth, J.; Boath, E.; Henshaw, C.; Brown, H. Department of S. Exercise as an adjunct treatment for postpartum depression for women living in an inner city —A pilot study. *Health Care Women Int.* **2017**, *38*, 635–639. https://doi.org/10.1080/07399332.2017.1295049.	8. Pentland et al. 2022<br>9. Wang et al. 2024

META-ANALYSES FOCUSED ON AEROBIC EXERCISE

Note: The following reviews were not included in the overlap calculation:

- Deprato et al. 2025 (the authors did not include the references that were meta-analyzed in the list of references)
- Ji et al. 2024 (the authors did not specify the studies that were meta-analyzed)

$$CCA = \frac{N-r}{rc-r} = \frac{36-28}{56-28} \quad \frac{8}{28} = 0.28 = 28\%$$

Note: N is the total number of original studies (including duplicates) in the meta-analyses of interest (the sum of all checked boxes in the citation matrix). Furthermore, r is the number of original studies without accounting for duplicates. Finally, c is the number of systematic reviews included in the evidence matrix (k=2). CCA = corrected covered area.

Number of studies without accounting for duplicates	Primary research (references)	Systematic reviews where are included primary research
1.	Aguilar-Cordero, M.J.; Sánchez-García, J.C.; Rodriguez-Blanque, R.; Sánchez-López, A.M.; Mur-Villar, N. Moderate Physical Activity in an Aquatic Environment During Pregnancy (SWEP Study) and Its Influence in Preventing Postpartum Depression. *J. Am. Psychiatr. Nurses Assoc.* **2019**, *25*, 112–121. https://doi.org/10.1177/1078390317753675	1. Xu et al. 2023
2.	Armstrong, K.; Edwards, H. The effects of exercise and social support on mothers reporting depressive symptoms: A pilot randomized controlled trial. *Int. J. Ment. Health Nurs.* **2003**, *12*, 130–138	2. Pritchett et al. 2017

3.	Armstrong, K.; Edwards, H. The effectiveness of a pram-walking exercise programme in reducing depressive symptomatology for postnatal women. *Int. J. Nurs. Pract.* **2004**, *10*, 177–194. https://doi.org/10.1111/j.1440-172X.2004.00478.xx.	3. Xu et al. 2023
4.	Boath, E.; Henshaw, C.; Forsyth, J. Exercise as an adjunct therapy for postnatal depression: A pilot study. In Proceedings of the International Marcé Society for Perinatal Mental Health Biennial Scientific Conference, Adelaide, Australia, 15 February 2015.	4. Pritchett et al. 2017
5.	Buttner, M.M.; Brock, R.L.; O'Hara, M.W.; Stuart, S. Efficacy of yoga for depressed postpartum women: A randomized controlled trial. *Complement. Ther. Clin. Pract.* **2015**, *21*, 94–100. https://doi.org/10.1016/j.ctcp.2015.03.003.	5. Xu et al. 2023
6.	Coll, C.D.V.N.; Domingues, M.R.; Stein, A.; da Silva, B.G.C.; Bassani, D.G.; Hartwig, F.P. et al. Efficacy of regular exercise during pregnancy on the prevention of postpartum depression: The PAMELA Randomized Clinical Trial. *JAMA Netw. Open* **2019**, *2*, e186861. https://doi.org/10.1001/jamanetworkopen.2018.6861.	6. Xu et al. 2023
7.	Da Costa, D.; Lowensteyn, I.; Abrahamowicz, M.; Ionescu-Ittu, R.; Dritsa, M.; Rippen, N.; Cervantes, P.; Khalifé, S. A randomized clinical trial of exercise to alleviate postpartum depressed mood. *J. Psychosom. Obstet. Gynecol.* **2009**, *30*, 191–200. .	7. Pritchett et al. 2017<br>8. Xu et al. 2023
8.	Daley, A.J.; Winter, H.; Grimmett, C.; McGuinness, M.; McManus, R.; MacArthur, C. Feasib-ility of an exercise intervention for women with postnatal depression: A pilot randomised controlled trial. *Br. J. Gen. Pract.* **2008**, *58*, 178–183. https://doi.org/10.3399/bjgp08x277195.	9. Pritchett et al. 2017<br>10. Xu et al. 2023
9.	Daley, A.J.; Blamey, R.V.; Jolly, K.; Roalfe, A.K.; Turner, K.M.; Coleman, S.; McGuinness, M.; Jones, I.; Sharp, D.J.; MacArthur, C. A pragmatic randomized controlled trial to evaluate the effectiveness of a facilitated exercise intervention as a treatment for postnatal depression: The PAMPeRS trial. *Psychol. Med.* **2015**, *45*, 2413–2425	11. Pritchett et al. 2017<br>12. Xu et al. 2023
10.	Forsyth, J.; Boath, E.; Henshaw, C.; Brown, H. Department of S. Exercise as an adjunct treatment for postpartum depression for women living in an inner city —A pilot study. *Health Care Women Int.* **2017**, *38*, 635–639. https://doi.org/10.1080/07399332.2017.1295049.	13. Xu et al. 2023
11.	Haruna, M.; Watanabe, E.; Matsuzaki, M.; Ota, E.; Shiraishi, M.; Murayama, R.; Yoshida, M.; Yeo, S. The effects of an exercise program on health-related quality of life in postpartum mothers: A randomised controlled trial. *Health* **2013**, *5*, 432–439.	14. Pritchett et al. 2017<br>15. Xu et al. 2023
12.	Heh, S.S.; Huang, L.H.; Ho, S.M.; Fu, Y.Y.; Wang, L.L. Effectiveness of an exercise support program in reducing the severity of postnatal depression in Taiwanese women. *Birth* **2008**, *35*, 60–65. https://doi.org/10.1111/j.1523-536X.2007.00192.x.	16. Xu et al. 2023
13.	Huang, L.; Li, L.X.; Xu, S.L.; Zang, F.D.; Gao, M.F. The effect of perinatal psychological c-ounseling and exercise training on postnatal rehabilitation. *Chin. J. Nurs.* **2003**, *38*, 28–30.	17. Xu et al. 2023

14.	Keller, C.; Ainsworth, B.; Records, K.; Todd, M.; Belyea, M.; Vega-López, S.; Permana, P.; Coonrod, D.; Nagle-Williams, A. A comparison of a social support physical activity intervention in weight management among post-partum Latinas. *BMC Public Health* **2014**, *14*, 971.	18. Pritchett et al. 2017<br>19. Xu et al. 2023
15.	Lewis, B.A.; Schuver, K.; Dunsiger, S.; Samson, L.; Frayeh, A.L.; Terrell, C.A.; Ciccolo, J.T.; Avery, M.D. Rationale, design, and baseline data for the Healthy Mom II Trial: A randomized trial examining the efficacy of exercise and wellness interventions for the prevention of postpartum depression. *Contemp. Clin. Trials* **2018**, *70*, 15–23. https://doi.org/10.1016/j.cct.2018.05.002	20. Xu et al. 2023
16.	Li, L.; Gao, L. Effects of yoga combined with psychological counseling on mental health and recovery body type and pelvic floor function recovery of primiparas with postpartum depression. *China J. Health Psychol.* **2019**, *27*, 118–123. https://doi.org/10.13342/j.cnki.cjhp.2019.01.035. (In Chinese)	21. Xu et al. 2023
17.	Mohammadi, F.; Malakooti, J.; Babapoor, J.; Mohammad-Alizadeh-Charandabi, S. The effe-ct of a homebased exercise intervention on postnatal depression and fatigue: A randomized controlled trial. *Int. J. Nurs. Pract.* **2015**, *21*, 478–485. https://doi.org/10.1111/ijn.12259	22. Xu et al. 2023
18.	Norman, E.; Sherburn, M.; Osborne, R.H.; Galea, M.P. An exercise and education program improves well-being of new mothers: A randomized controlled trial. *Phys. Ther.* **2010**, *90*, 348–355	23. Pritchett et al. 2017<br>24. Xu et al. 2023
19.	Ozkan, S.A.; Kucukkelepce, D.S.; Korkmaz, B.; Yılmaz, G.; Bozkurt, M.A. The effectiveness of an exercise intervention in reducing the severity of postpartum depression: A randomized controlled trial. *Perspect. Psychiatr. Care* **2020**, *56*, 844–850. https://doi.org/10.1111/ppc.12500	25. Xu et al. 2023
20.	Ren, W.; Wu, C.W.; Xie, Y. Effects of moderate-intensity aerobic exercise on mild to moderate postpartum depression and self-efficacy. *Mod. J. Integr. Tradit. Chin. West. Med.* **2019**, *28*, 1500–1503. https://doi.org/10.3969/j.issn.1008-8849. (In Chinese)<br>.	26. Xu et al. 2023
21.	Robichaud, K.P. The Effects of an Exercise Intervention on the Psychological Well-Being of Postpartum Women. Ph.D. Thesis, Middle Tennessee State University, Murfreesboro, TN, USA, 2008. Available online: http://jewlscholar.mtsu.edu/bitstream/ handle/mtsu/4064/3322482.pdf?sequence=1&isAllowed=y (accessed on 3 August 2017	27. Pritchett et al. 2017<br>28. Xu et al. 2023
22.	Saeedi, S. Effect of exercise program on symptoms of postpartum depression. *Iran. J. Obstet. Gynecol. Infertil.* **2013**, *15*, 26–31. https://doi.org/10.22038/IJOGI.2013.287	29. Xu et al. 2023
23.	Shelton, S. Postpartum Depressive Symptoms: A Study of Influencing Factors and an Int-Ervention for Improvement. Ph.D. Thesis, Georgia State University, Atlanta, GA, USA, 2015. [111]	30. Xu et al. 2023
24.	Surkan, P.J.; Gottlieb, B.R.; McCormick, M.C.; Hunt, A.; Peterson, K.E. Impact of a health promotion intervention on maternal depressive symptoms at 15 months postpartum. *Matern. Child Health J.* **2012**, *16*, 139–148. https://doi.org/10.1007/s10995-010-0729-x.<br>.	31. Xu et al. 2023
25.	Teychenne, M.; Abbott, G.; Stephens, L.D.; Opie, R.S.; Olander, E.K.; Brennan, L.; van der Pligt, P.; Apostolopoulos, M.; Ball, K. Mums on the move: A pilot randomised controlled trial of a home-based physical activity intervention for mothers at risk of postnatal depression. *Midwifery* **2021**, *93*, 102898. https://doi.org/10.1016/j.midw.2020.102898.	32. Xu et al. 2023

26.	Thiruppathi, A.; Prasana, B.; Mastaniah, E.; Vamsidhar, N.; Himabindu, P. A structured physical activity and health care education beats postpartum depression for primipara mothers: A pilot randomized controlled trial. *Int. J. Physiother.* **2014**, *1*, 144–151.	33. Pritchett et al. 2017<br>34. Xu et al. 2023
27.	Yan, F. A Study of the Effect of a Fertility Dance Intervention on Perinatal Depression in Advanced Postpartum Menstrual Age. Southern Medical University, Guangzhou, China, 2019. https://doi.org/10.27003/d.cnki.gojyu.2019.000068.	35. Xu et al. 2023
28.	Yang, C.L.; Chen, C.H. Effectiveness of aerobic gymnastic exercise on stress, fatigue, and sleep quality during postpartum: A pilot randomized controlled trial. *Int. J. Nurs. Stud.* **2018**, *77*, 1–7. https://doi.org/10.1016/j.ijnurstu.2017.09.009.	36. Xu et al. 2023

META-ANALYSES FOCUSED ON MULTIMODAL EXERCISE PROGRAMS

Note: The following reviews were not included in the overlap calculation:

- Deprato et al. 2025 (the authors did not include the references that were meta-analyzed in the list of references)
- Ji et al. 2024 (the authors did not specify the studies that were meta-analyzed)

THE DEGREE OF OVERLAP COULD NOT BE CALCULTAED
